# Human papillomavirus detected in female breast carcinomas in Japan

**DOI:** 10.1038/sj.bjc.6604502

**Published:** 2008-07-22

**Authors:** N A Khan, A Castillo, C Koriyama, Y Kijima, Y Umekita, Y Ohi, M Higashi, Y Sagara, H Yoshinaka, T Tsuji, S Natsugoe, T Douchi, Y Eizuru, S Akiba

**Affiliations:** 1 Department of Epidemiology and Preventive Medicine, Kagoshima University Graduate School of Medical and Dental Sciences, 8-35-1 Sakuragaoka, Kagoshima 890-8544, Japan; 2 Department of Surgical Oncology, Breast and Endocrine Surgery, Kagoshima University Graduate School of Medical and Dental Sciences, 8-35-1 Sakuragaoka, Kagoshima 890-8544, Japan; 3 Department of Tumor Pathology, Kagoshima University Graduate School of Medical and Dental Sciences, 8-35-1 Sakuragaoka, Kagoshima 890-8544, Japan; 4 Sagara Hospital, 3-31, Matsubara, Kagoshima 892-0833, Japan; 5 Department of Human Pathology, Kagoshima University Graduate School of Medical and Dental Sciences, 8-35-1 Sakuragaoka, Kagoshima 890-8544, Japan; 6 Department of Reproductive Pathophysiology and Obstetrics-Gynecology, Kagoshima University Graduate School of Medical and Dental Sciences, 8-35-1 Sakuragaoka, Kagoshima 890-8544, Japan; 7 Division of Persistent and Oncogenic Viruses, Center for Chronic Viral Diseases, Kagoshima University Graduate School of Medical and Dental Sciences, 8-35-1 Sakuragaoka, Kagoshima 890-8544, Japan

**Keywords:** human papillomavirus, breast cancer, HPV-16, viral load, physical status

## Abstract

To investigate the aetiological role of human papillomavirus (HPV) in breast cancer, we examined the presence, genotype, viral load, and physical status of HPV in 124 Japanese female patients with breast carcinoma. Human papillomavirus presence was examined by PCR using SPF10 primers, and primer sets targeting the *E6* region of HPV-16, -18, and -33. The INNO-LiPA HPV genotyping kit was used to determine genotype. Human papillomavirus DNA was detected in 26 (21%) breast carcinomas. The most frequently detected HPV genotype was HPV-16 (92%), followed by HPV-6 (46%), HPV-18 (12%), and HPV-33 (4%). In 11 normal epithelium specimens adjacent to 11 HPV-16-positive carcinomas, 7 were HPV-16-positive. However, none of the normal breast tissue specimens adjacent to HPV-negative breast carcinomas were HPV-positive. The real-time PCR analysis suggested the presence of integrated form of viral DNA in all HPV-16-positive samples, and estimated viral load was low with a geometric mean of 5.4 copies per 10^4^ cells. In conclusion, although HPV DNA was detected in 26 (21%) breast carcinomas and, in all HPV-16-positive cases, the HPV genome was considered integrated into the host genome, their low viral loads suggest it is unlikely that integrated HPV is aetiologically involved in the development of Japanese breast carcinomas that we examined.

Breast cancer is one of the most prevalent malignancies throughout the world. It is the leading female cancer worldwide and comes second after stomach cancer in Japan (Parkin *et al*, 1999). Recent studies have suggested that some types of viruses, especially human papillomavirus (HPV), might be involved in the pathogenesis of breast cancer (Liu *et al*, 2001; Tsai *et al*, 2007).

Molecular and epidemiological studies have shown that a persistent infection with high-risk HPV is the most important risk factor for both cervical cancer and its precursors (Cuschieri *et al*, 2005; Steenbergen *et al*, 2005). A number of studies have reported HPV DNA detection in extragenital cancers, although the aetiological involvement of HPV in those malignancies is still controversial (Gillison and Shah, 2003). Recently, it has been shown that HPV types 16 and 18 can immortalize normal breast epithelium (Band *et al*, 1990; Wazer *et al*, 1995). This raised the possibility that HPV may be aetiologically related to some cases of breast cancer. However, unlike cervical carcinoma, which is almost always associated with HPV, the causal role of HPV infection in the development of breast carcinoma remains controversial.

Di Lonardo *et al* (1992) were the first to report the relationship between HPV and breast cancer, demonstrating HPV-16 DNA in 29.4% of breast carcinomas, using PCR with primer sets specific for HPV-11, -16, and -18 genotypes. A study in Norway, which used primers specific for HPV-11, -16, -18, and -33, in addition to GP5+/6+ primers, detected HPV in 46% of breast carcinomas. In this previous study, only HPV-16 was detected (Hennig *et al*, 1999). Another European study showed the presence of high-risk HPVs in 15% of invasive breast carcinomas in Greece (Kroupis *et al*, 2006). A Chinese study also found that 35% of breast carcinomas examined were HPV-16-positive, using type-specific primers for HPV-16 and consensus primers for the HPV *L1* gene (Liu *et al*, 2001). In addition, a study in Korea reported HPV DNA presence in breast carcinomas (6.5%) but not in intraductal papilloma (Choi *et al*, 2007). Furthermore, a study in Brazil demonstrated HPV-16 and HPV-18 in 14 and 10% of breast carcinomas, respectively, using genotype-specific primers (Damin *et al*, 2004). In addition, an Australian study reported by Kan *et al* (2005) used primers specific for HPV-16, -18, and -33, and demonstrated the presence of HPV-18 in 48% of breast cancer specimens. However, neither HPV-16 nor HPV-33 was detected in their study. Another study conducted in China and Japan, which used primers for HPV-16, -18, and -33, detected HPV-33 in 41.7 and 11.1% of breast carcinomas in two countries, respectively (Yu *et al*, 1999). By examining nipple and breast cancer specimens, de Villiers *et al* (2005) detected high-risk and low-risk HPVs in 86% of them. They used GP5+/6+, FAP primers for cutaneous-type HPVs, and CP primers. In contrast, several other studies failed to demonstrate HPV DNA in tumors of the breast (Wrede *et al*, 1992; Lindel *et al*, 2007).

In malignant transformation of the uterine cervical epithelia, the integration of high-risk HPV DNA into the host genome is considered an important step (zur Hausen, 1991). Viral DNA integration leads to frequent disruption of the HPV-16 *E2* gene (Kalantari *et al*, 1998), a negative regulator of the *E6*/*E7* promoter, resulting in the upregulation of the transcription of the *E6* and *E7* oncogenes. Human papillomavirus-16 *E6* and *E7* oncogenes interfere with the normal cell cycle by targeting the p53 and pRb tumor suppressor proteins, respectively (Vousden, 1993). Human papillomavirus-16 DNA integration into the host genome possibly provides a selective growth advantage to the host cell (Jeon *et al*, 1995; Jeon and Lambert, 1995).

When considering the aetiological role of HPV in non-genital cancers, an important question that should be addressed is the integration of HPV into the host genome. Venuti *et al* (2000) found that 43% of HPV-16-positive laryngeal carcinomas had HPV-16 DNA integrated into the host genome. Koskinen *et al* (2003) also reported that 65% of HPV-16-positive head and neck carcinomas had integrated HPV-16 DNA. Recent studies conducted by our laboratory also showed that most HPV-16-positive esophageal and lung carcinomas had integrated HPV-16 DNA (Aguayo *et al*, 2007; Shuyama *et al*, 2007). To our knowledge, however, none of the published studies have investigated the possibility of integration of HPV in breast cancer tissues.

Considering the controversial reports on the aetiology of HPV in breast carcinomas, the aim of this study was to examine the presence, genotype, viral load, and physical status of HPV, particularly high-risk HPV, among breast carcinomas in Japan, where breast cancer risk is low when compared with Europe and North America (Parkin *et al*, 2002), and to investigate its aetiological role.

## Materials and methods

### Study subjects

A total of 124 formalin-fixed and paraffin-embedded breast cancer specimens consisting of 42 cases from Sagara Hospital (Kagoshima, Japan), diagnosed during the period between 2000 and 2001, and 82 cases from Kagoshima University Hospital (Kagoshima, Japan), diagnosed during the period between 2000 and 2006, were examined in this study. In addition, as positive controls, we examined formalin-fixed and paraffin-embedded specimens from six cervical cancer cases diagnosed at Kagoshima University Hospital. Clinicopathological information, including the expression of oestrogen and progesterone receptors, reproductive histories, and family history of breast cancer, was obtained from medical and pathological records. Histological classification for breast cancer was made using the guidelines determined by the Japanese Breast Cancer Society (2004). With regard to cervical cancer, we also applied the guidelines determined by the Japan Society of Obstetrics and Gynaecology, The Japanese Society of Pathology, and the Japan Radiological Society (1999). The Institutional Review Board of Kagoshima University Graduate School of Medical and Dental Sciences (Japan) approved this study.

### DNA extraction

Sections of 10 *μ*m thickness were cut and collected in sterile tubes. To avoid contamination between samples, a cutter blade was changed between each sample cutting. For DNA extraction from paraffin-embedded tissue, each sample was treated with 0.8 ml of lemosol and 0.2 ml of ethanol, and subsequently washed with 1 ml of ethanol. After centrifugation and air-drying, the pellet was resuspended in digestion buffer (50 mM Tris-HCl pH 8.0, 1 mM EDTA, pH 8.0, and 0.5% Tween 20) containing 200 *μ*g ml^−1^ of proteinase K (Invitrogen Corp., Carlsbad, CA, USA) and incubated for 24 h at 56°C. The solution was then heated at 100°C for 10 min, followed by phenol–chloroform extraction and ethanol precipitation of DNA. DNA quality and the absence of PCR inhibitors in samples were tested by PCR for *β-globin* using PCO3 5′-ACACAACTGTGTTCACTAGC-3′ and PCO4 5′-CAACTTCATCCACGTTCACC-3′ primers under the following PCR conditions: initial denaturation at 95°C for 15 min, 40 cycles with the cycling profile of 95°C for 1 min, 52°C for 1 min, 72°C for 1 min, and final extension for 5 min at 72°C.

### HPV detection and genotyping

The HPV genome was detected with a broad-spectrum SPF10-biotinylated primer PCR. The PCR products, 65 bp of the *L1* gene, were run on a 4% agarose gel and visualized with ethidium bromide staining by electrophoresis. Human papillomavirus typing of the HPV DNA-positive samples was performed using the INNO-LiPA HPV Genotyping v2 test (Innogenetics, Ghent, Belgium) (Ketler *et al*, 1999). In addition, we also analysed all samples through additional type-specific PCR using primer sets targeting the *E6* regions of HPV-18 and HPV-33 (Yu *et al*, 1999). These type-specific primers could detect 140 bp in the *E6* region of HPV-18 and HPV-33.

Paraffin sections without tissue and distilled water were used as negative controls for procedures of DNA extraction and PCR, respectively. Full genomes of HPV-16 and HPV-18 cloned in pUC19 plasmid (kindly given by Dr Massimo Tommasino, IARC, Lyon, France), and HPV-33-positive cervical cancer specimens were used as positive controls for amplification.

### Quantitative real-time PCR

To determine the presence and physical status, as well as estimate the viral load of HPV-16, real-time PCR was performed with the ABI Prism 7700 Sequence Detection System (Applied Biosystems, Foster City, CA, USA) and 2 × QuantiTect SYBR Green PCR kit (Qiagen, Hilden, Germany). The HPV-16 primers for *E6* ORF amplification were as follows: *E6*F: 5′-GAGAAACTGCAATGTTTCAGGACC-3′ and *E6*R: 5′-TGTATAGTTGTTTGCAGCTCTGTGC-3′. The HPV-16 primers for *E2* ORF amplification were as follows: *E2*F: 5′-AACGAAGTATCCTCTCCTGAAATTATTAG-3′ and *E2*R: 5′-CCAAGGCGACGGCTTTG-3′. The PCR amplification conditions were 2 min at 50°C, 15 min at 95°C, and a two-step cycle of 95°C for 15 s, and 60°C for 60 s for a total of 40 cycles (Peitsaro *et al*, 2002). The sizes of the *E6* and *E2* products were 81 and 76 bp, respectively.

Serial dilutions of the full-length HPV-16 genome, containing equivalent amounts of *E2* and *E6* genes from 86 to 862 million copies per reaction, served as a standard control. Numbers of the threshold cycle obtained from *E2* PCR and *E6* PCR were equivalent in each run. Linear plots of the log of copy number *vs* numbers of threshold cycle were consistently obtained for both genes.

Real-time PCR for the *β-globin* gene was performed by the 2 × QuantiTect SYBR Green PCR kit (Qiagen) using PC03/PC04 to adjust the differences in the amount of input genomic DNA between samples. A seven-fold dilution series of a human DNA control (Dynal, UK Ltd, Bromborough, Wirral, Merseyside, UK) was used to generate the standard curve. The amount of *β-globin* DNA present in each sample was divided by the weight of one genome equivalent (that is, 6.6 pg per cell) and a factor of 2 (as there are two copies of *β-globin* DNA/genome equivalent or cell) to obtain the number of genome equivalents or cell in the sample. Viral loads in each specimen were expressed as the number of HPV copies per 10^4^ cells.

### Immunohistochemistry for HER2/*neu* overexpression

HER2/*neu* overexpression was examined, using the Hercep Test kit (Dako, Carpinteria, CA, USA) following the manufacturer's instructions. Control cell lines provided by the manufacturer (Dako) were used as negative and positive controls. The data on its oevererexpression for 17 HPV-positive breast carcinomas (16 invasive carcinomas and 1 non-invasive carcinoma) and 42 HPV-negative carcinomas (40 invasive carcinomas and 2 non-invasive carcinoma) were obtained from medical records. In the HPV-positive cases, HER2/*neu* expression was additionally examined in seven cases. Following the Hercep Test criteria, the immunoreaction was scored as follows: 3+, complete and intense membrane staining of >10% tumor cells; 2+, complete but moderate staining of >10% tumor cells; 1+, weak and incomplete staining in >10% tumor cells; and 0, no membrane staining or staining in <10% tumor cells. We defined cases with a score 3+ as HER2/*neu* overexpression.

### Statistical analysis

Fisher's exact test was conducted to test statistical significance using STATA (version 8.5, Stata Corporation, College Station, TX, USA). All the *P*-values presented are two-sided.

## Results

We examined 124 female breast cancer cases, aged from 23 to 90 years with a mean of 55 years. *β-globin* amplification was carried out to check the quality of DNA in formalin-fixed and paraffin-embedded specimens. All samples were positive for *β-globin* gene amplification, indicating that DNA was available for molecular analysis.

First, we examined all samples using novel, broad-spectrum SPF10 primers for HPV detection. Human papillomavirus DNA was positive in 24 out of 124 (19%) breast cancer specimens. As an alternative approach, we examined all specimens using primer sets targeting the *E6* region of HPV genotypes 16, 18, and 33. By using genotype-specific primers, HPV-16 was detected in two new cases. No additional cases of HPV-18 and HPV-33 were detected by genotype-specific approach. Thus, in total, HPV DNA was detected in 26 (21%) breast cancer specimens.

Table 1 presents the HPV frequency according to various clinicopathological features. There was no significant difference in the HPV detection rate between invasive and non-invasive carcinomas. Among invasive carcinomas, HPV DNA was detected in 7 (20%) papillotubular carcinomas, 5 (29%) solid-tubular carcinomas, and 9 (20%) scirrhous carcinomas. Human papillomavirus detection rates were not different among those histological types of invasive carcinomas (data not shown in the table). The presence of HPV was not related to the expression of oestrogen and progesterone receptors. Among the 12 patients with bilateral breast carcinomas, we could obtain breast cancer specimens from the other side from 2 HPV-positive and 2 HPV-negative cases. We also examined the presence of HPV DNA in these specimens by the same PCR methods. However, none of them was HPV-positive.

HER2/*neu* expression was examined in 23 invasive and 1 non-invasive HPV-positive breast carcinomas. Three (13%) invasive carcinomas had its overexpression (score of 3+) and the non-invasive carcinoma had no HER2/*neu* overexpression (score=1). On the other hand, in HPV-negative carcinomas, its overexpression was observed in 6 out of 40 (15%) invasive carcinomas, and in 1 out of 2 non-invasive carcinomas. Neither in HPV-positive nor in HPV-negative carcinomas HER2/*neu* overexpression was associated with any clinicopathological factors, including tumor size, lymph vessel invasion, vascular invasion, and lymph node metastasis (data not shown).

The most prevalent HPV type was HPV-16, which was detected in 24 (92%) out of 26 HPV-positive cases, using the INNO-LiPA HPV genotyping assay and the type-specific approach (Table 2). Among them, three cases (BC-72, BC-78, and BC-112) were identified as HPV-16-positive only by the PCR with HPV-16-specific primers. Other HPV types, identified only by the INNO-LiPA HPV genotyping assay, were HPV-6 in 12 (50%) cases, HPV-18 in 3 (12%) cases, HPV-51 in 2 (8%) cases, and HPV-33 in 1 (4%) case. Multiple infections were found in 12 (46%) HPV-positive cases (Table 2).

All of the HPV-16-positive breast carcinoma specimens were subjected to quantitative real-time PCR analysis to determine the viral load and physical status of HPV-16. The HPV-16 copy numbers of *E6* and *E2* genes per 10^4^ cells, the ratios of *E2* to *E6*, and the viral physical status are compiled in Table 2. The geometric mean of HPV-16 copies per 10^4^ cells was 5.4 in cancers of the breast and 130 480 in cancers of the uterine cervix. In 19 (95%) breast carcinoma cases, *E2* DNA was not detected, and in 1 (5%) case, *E2* DNA was detected, but the *E2*/*E6* ratio was less than unity. In cervical carcinoma cases, there were two (33%) cases where *E2* DNA was not detected. On the other hand, there were four (67%) cases where the *E2* DNA was detected, but the *E2*/*E6* ratio was less than unity.

For 11 HPV-16-positive breast carcinoma specimens, we examined the presence of HPV DNA in 1 specimen of morphologically normal breast tissue adjacent to the carcinoma. Human papillomavirus-16 DNA was detected in seven (64%) of these specimens (Table 3). The geometric mean of viral load in the normal tissue was 92 copies per 10^4^ cells, which was higher than that in breast carcinomas (5.4 copies per 10^4^ cells); however, the difference was not statistically significant (*P*=0.162 by the Mann–Whitney test). The analysis of the *E2*/*E6* ratio in normal epithelium specimens showed that in one (17%) case, *E2* DNA was not detected, and in four (66%) cases, *E2* DNA was detected but the *E2*/*E6* ratio was less than unity. The remaining one (17%) case had an *E2*/*E6* ratio of approximately one. We also examined 11 normal breast specimens adjacent to HPV-negative breast tumor tissue, but none of them was HPV-positive (Table 3). The difference in HPV detection rates in surrounding normal mucosa of HPV-positive and HPV-negative breast carcinoma cases was statistically significant (*P*=0.004 by Fisher's exact test).

## Discussion

In this study, we found HPV DNA in 21% (*n*=26) of breast carcinomas in Japan. Another study conducted in Japan detected HPV DNA in 11.1% of breast carcinomas (Yu *et al*, 1999). High-risk types, such as HPV-16, -18, and -33, were detected in our study. Despite great variability in the HPV detection rate worldwide, the majority of HPV types that are detected are the oncogenic types, HPV-16 and -18 (Di Lonardo *et al*, 1992; Hennig *et al*, 1999; Liu *et al*, 2001; Damin *et al*, 2004; Kan *et al*, 2005). Our results are in agreement with these previous reports. The association of high-risk HPV was reported to be stronger in invasive carcinomas (Yasmeen *et al*, 2007b). In this study, however, the HPV frequency in invasive carcinomas was higher than that of non-invasive carcinomas although the difference was not statistically significant (*P*=0.279).

The physical status of HPV-16 was determined by the HPV-16 *E2*/*E6* ratio in this study. When the ratio was equal to or higher than unity, all of the HPV-16 genome was considered to be in the episomal form. Note, however, the *E2*/*E6* ratio of unity or larger does not necessarily deny the possibility of HPV integration because HPV integration does not always cause *E2* disruption (Vernon *et al*, 1997; Eriksson *et al*, 1999). However, as we found no breast carcinoma with the exclusive presence of episomal HPV-16 (*E2*/*E6* ratio being equal to or higher than unity), this assumption does not affect the interpretation of our observations. On the other hand, the lack of amplified HPV-16 *E2* genome was considered to indicate the integration of all of the HPV-16 DNA into the host genome. When the *E2*/*E6* ratio was larger than zero and smaller than unity, this condition was considered as a mixture of episomal and integrated forms (Peitsaro *et al*, 2002). We found HPV-16 DNA integrated into the host genome in all HPV-16-positive breast carcinoma cases, although one case harboured both integrated and episomal forms of HPV-16. In cervical cancer samples, 67% of the samples had HPV-16 in a mixed form, where HPV existed in both episomal and integrated forms, and 33% of the samples had only the integrated form of HPV. To our knowledge, this is the first study reporting the physical status of HPV-16 in breast cancer tissues.

In this study, the HPV-16 viral load determined by quantitative real-time PCR in breast carcinoma was with a geometric mean of 5.4 per 10^4^ cells, which was much lower than that observed in cervical cancer (geometric mean=130480). It is unlikely, however, that the low viral loads in breast carcinoma specimens were a consequence of formalin fixation and/or heating during paraffin embedding, as a higher viral load was detected in formalin-fixed and paraffin-embedded specimens of cervical cancer obtained from the same institute. Moreover, we performed parallel amplification of a housekeeping gene (*β-globin*) to obtain an estimate of the amount of amplifiable genomic DNA in each sample, and to help reconcile the inhibitory effects of formalin fixation and paraffin embedding on DNA amplification.

A low copy number of HPV-16 in non-genital carcinomas has also been reported in studies of Chilean lung carcinomas (Aguayo *et al*, 2007) and Chinese oesophageal cancer (Shuyama *et al*, 2007) conducted by our laboratory, and in a Finish study on head and neck cancer (Koskinen *et al*, 2003). It should also be noted that SiHa cells, which are a cervical cancer cell line, contain only 1–2 copies of HPV-16 per cell. Such a low copy number of HPV DNA in SiHa cells suggests that a viral load as low as 1 copy per cell may be sufficient to promote carcinogenesis, especially if the viral DNA is integrated into the host cell genome (Si *et al*, 2005). In this study, however, the viral load in breast carcinoma was much lower than 1 copy per cell. As breast cancer is considered a monoclonal expansion and the integrated HPV genome is unlikely to disappear from the host genome during cell replication, at least one copy of integrated HPV is expected to be present in all carcinoma cells even if only a copy of integrated HPV is sufficient for its aetiological involvement. Therefore, even though HPV-16 was found integrated in breast cancer cells in this study, its extremely low viral load suggests that HPV-16 DNA is unlikely to be involved in the development of those breast carcinomas.

In this study, HPV-16 was also found in 64% of normal mucosal specimens adjacent to HPV-16-positive tumors but not in the non-malignant epithelia from HPV-16-negative tumors. The presence of HPV-16, not only in breast carcinomas but also in adjacent normal tissue, can also be explained by the infection of HPV in carcinomas after their development because both carcinoma and adjacent normal tissue are likely to be exposed to such an infection.

Hormonal factors (namely, oestrogen, and its derivatives) are known to be involved in breast carcinogenesis (Pike *et al*, 1993). Oestrogen receptor-positive breast carcinomas are more sensitive to tamoxifen, an antioestrogen. An interesting question is whether oestrogen-related breast cancer is strongly related to HPV. In this regard, Hennig *et al* (1999) and Damin *et al* (2004) reported that the presence of oestrogen or progesterone receptor was not related to HPV presence in breast carcinomas. We also did not observe any significant association of HPV presence with oestrogen and progesterone receptor expression.

Approximately 25–30% of all breast cancers have an amplification of the HER2/*neu* gene or overexpression of its protein product (Slamon *et al*, 1987). Yasmeen *et al* (2007a) reported that *E6*/*E7* of HPV-16 cooperates with the HER2/*neu* receptor to induce breast tumorigensis and metastasis. This study, however, did not show any evidence indicating that HPV presence affects the relationship between HER2/*neu* overexpression and tumor invasiveness.

We did not find any difference in the average age at diagnosis between HPV-positive and HPV-negative breast cancers. Similar results were described in studies of Norwegian and Brazilian women (Hennig *et al*, 1999; Damin *et al*, 2004). In contrast, Kroupis *et al* (2006) and Lawson *et al* (2006) demonstrated that the average age of women with HPV-positive breast cancer is significantly younger than women with HPV-negative breast cancer, suggesting that sexual activity is one of the risk factors of HPV-positive breast cancer.

In cervical cancer, the cytopathic effect of HPV infection is well known as koilocytosis. There are several studies reporting similar histological features among non-genital cancers (Miller *et al*, 1997; Al-Qahtani *et al*, 2007). Although we reviewed haematoxylin–eosin staining slides of all HPV-positive cases, none of them showed pathological features of HPV infection such as koilocytes (data not shown).

The transmission route of HPV detected in breast carcinoma is yet unclear. Two independent studies suggested possible hematogenic and/or lymphatic transfer of the virus from one organ to another (Hennig *et al*, 1999; Widschwendter *et al*, 2004). Interestingly, the study by Hennig *et al* (1999) detected HPV-16 DNA in 46% of breast cancer occurring among women with a history of high-grade cervical intraepithelial neoplasia (CIN III). Their finding suggests that HPV-associated cervical neoplasia might be the original site of HPV infection from which the virus could be transported to the breast. In our study, there were four breast cancer cases with a history of cervical cancer but only 1 of them was HPV-positive in their breast tumors. de Villiers *et al* (2005) showed HPV presence in the nipple, suggesting HPV transfer in a retrograde fashion from the nipple via areola, lactiferous ducts, and sinuses.

The reported prevalence of HPV infection in breast cancer shows a great variation worldwide, ranging from 0 to 86% (Hennig *et al*, 1999; Damin *et al*, 2004; de Villiers *et al*, 2005; Kan *et al*, 2005; Kroupis *et al*, 2006; Choi *et al*, 2007; Lindel *et al*, 2007; Yasmeen *et al*, 2007b). Demographic features and genetic backgrounds may contribute to the geographical difference of HPV prevalence in breast carcinomas worldwide. In addition, the difference in published reports may be attributed to the numbers of samples tested, methodological differences, and the sensitivity of methods used, such as use of different primer sets. In this study, the use of HPV-16-specific primers detected three additional HPV-16-positive cases, which were HPV-16-negative by SPF10-PCR, although the SPF10 primer set is considered highly sensitive. The discrepancy between the SPF10 consensus primer set PCR and PCR with HPV-16-specific primers stems from the fact that SPF10 primers target the sequence of the *L1* gene, which may be lost during the integration of HPV-16 into the host genome (Noffsinger *et al*, 1995; Karlsen *et al*, 1996).

In summary, HPV DNA was detected in 26 (21%) breast carcinomas and, in all HPV-16-positive cases, the HPV genome was considered to be integrated into the host genome. However, their extremely low viral loads suggest that it is unlikely that integrated HPV-16 is aetiologically involved in the development of Japanese breast cancer cases examined in this study. As reported by Shuyama *et al* (2007), the role of HPV in non-genital cancer may vary in different study areas. Therefore, in the case of breast cancer, we cannot deny the possibility that HPV may play a significant role in breast cancer development in other countries. Further studies are warranted to elucidate the aetiological role and pathogenesis of HPV in breast cancer.

## Figures and Tables

**Table 1:** Frequency of HPV-positive cases by clinicopathological factors

	**All cases**	**HPV-negative**	**HPV-positive**	
	***N* (%)**	***N* (%)**	***N* (%)**	***P*-value^a^**
Number of cases	124 (100)	98 (79)	26 (21)	
				
*Tumor depth*				0.279
Non-invasive	25 (100)	22 (88)	3 (12)	
Invasive	99 (100)	76 (77)	23 (23)	
				
*Age (years)*				0.966
<40	15 (100)	12 (80)	3 (20)	
40–49	37 (100)	28 (76)	9 (24)	
50–59	27 (100)	22 (81)	5 (19)	
60–	45 (100)	36 (80)	9 (20)	
				
*Family history of breast cancer* b				1.000
No	110 (100)	87 (79)	23 (21)	
Yes	12 (100)	10 (83)	2 (17)	
				
*Have married* b				1.000
No	7 (100)	6 (86)	1 (14)	
Yes	115 (100)	91 (79)	24 (21)	
				
*Number of children* b				0.613
None	22 (100)	19 (86)	3 (14)	
1	15 (100)	13 (87)	2 (13)	
2	49 (100)	36 (73)	13 (27)	
3–	35 (100)	28 (80)	7 (20)	
				
*Tumor size*				0.126
			(*P* for trend =0.321)	
Tis or T1mic	28 (100)	24 (86)	4 (14)	
T1a	10 (100)	9 (90)	1 (10)	
T1b	16 (100)	14 (88)	2 (12)	
T1c	33 (100)	22 (67)	11 (33)	
T2	27 (100)	19 (70)	8 (30)	
T3−	10 (100)	10 (100)	0 (0)	
				
*Lymph vessel invasion*				0.612
No	93 (100)	72 (77)	21 (23)	
Yes	31 (100)	26 (84)	5 (16)	
				
*Vascular invasion* b				0.343
No	116 (100)	90 (78)	26 (22)	
Yes	7 (100)	7 (100)	0 (0)	
				
*Lymph node metastasis* b				1.000
No	99 (100)	78 (79)	21 (21)	
Yes	24 (100)	19 (79)	5 (21)	
				
*Oestrogen receptor expression* b				1.000
No	24 (100)	19 (79)	5 (21)	
Yes	88 (100)	68 (77)	20 (23)	
				
*Progesterone receptor expression* b				1.000
No	37 (100)	29 (78)	8 (22)	
Yes	74 (100)	57 (77)	17 (23)	
				
*Breast cancer at both sides* b				0.233
No	110 (100)	88 (80)	22 (20)	
Yes	12 (100)	8 (67)	4 (33)	

HPV=human papillomavirus.

a *P*-values for heterogeneity were obtained by Fisher's exact test.

b Total number of cases did not add up to 124 because the cases without relevant information were excluded from analysis.

**Table 2:** HPV genotypes by INNO-LiPA and HPV-16 viral load and physical status using real-time PCR in breast and cervical carcinoma

		**Viral load**		**Integration**	
**Sample**	**Genotype by INNO-LiPA**	***E6* copies per 10^4^ cells**	***E2* copies per 10^4^ cells**	***E2/E6***	**Status**
*Breast cancer*					
BC-4	6, 16, 51	10	0	No *E2*	Integrated
BC-18	6, 16	4	0	No *E2*	Integrated
BC-19	16	28	0	No *E2*	Integrated
BC-27	6, 16	97	0	No *E2*	Integrated
BC-35	6, 16, 33, 51	3	0	No *E2*	Integrated
BC-42^a^	6, 16				
BC-44	6, 16	7184	0	No *E2*	Integrated
BC-60	6, 16	19	0	No *E2*	Integrated
BC-61	6, 18				
BC-68	16	6	0	No *E2*	Integrated
BC-69	16	648	32	0.05	Mixed
BC-70	16	31	0	No *E2*	Integrated
BC-72^b^		<1	0	No *E2*	Integrated
BC-78^b^	6	6	0	No *E2*	Integrated
BC-81	16	7	0	No *E2*	Integrated
BC-82	16	<1	0	No *E2*	Integrated
BC-92	16	3	0	No *E2*	Integrated
BC-95^a^	6, 16				
BC-100	16	<1	0	No *E2*	Integrated
BC-101^a^	6, 16				
BC-105	16	6	0	No *E2*	Integrated
BC-110	18				
BC-112^b^		<1	0	No *E2*	Integrated
BC-128^a^	6, 16, 18				
BC-131	16	4	0	No *E2*	Integrated
BC-132	16	<1	0	No *E2*	Integrated
					
*Cervical cancer*					
CX-2	16	8440	0	No *E2*	Integrated
CX-4	16	9 159 670	162 656	0.018	Mixed
CX-7	16, 18, 68	1328	0	No *E2*	Integrated
CX-10	6, 16	82 043	1181	0.014	Mixed
CX-11	6, 16	463 450	1420	0.003	Mixed
CX-13	16	1 264 077	17 343	0.014	Mixed

a The template DNAs could not be amplified by both *E6*- and *E2*-specific primers for HPV-16.

b These cases were HPV-16-negative by the INNO-LiPA genotyping kit.

**Table 3:** HPV genotypes by INNO-LiPA and HPV-16 viral load and physical status in surrounding normal epithelium of HPV-positive and HPV-negative breast carcinoma cases

		**Viral load**		**Integration**	
**Sample**	**Genotype by INNO-LiPA**	***E6* copies per 10^4^ cells**	***E2* copies per 10^4^ cells**	**E2/E6**	**Status**
*HPV-positive*					
N-4	Negative				
N-18	Negative				
N-68	16	301	133	0.44	Mixed
N-69	16	3206	32	0.05	Mixed
N-70	16	25	<1	0.025	Mixed
N-72	Negative				
N-78	16	14 972	0	No *E2*	Integrated
N-82	16	<1	<1	1	Episomal
N-100	Negative				
N-105	16	4	2	0.49	Mixed
N-112	16	a	a		
					
*HPV-negative*					
N-19	Negative				
N-50	Negative				
N-55	Negative				
N-57	Negative				
N-65	Negative				
N-66	Negative				
N-92	Negative				
N-94	Negative				
N-95	Negative				
N-102	Negative				
N-108	Negative				

HPV=human papillomavirus.

a There was not enough DNA template for this assay.
